# Kinase perturbations redirect mitochondrial function in cancer

**DOI:** 10.26124/bec:2022-0013

**Published:** 2022-11-15

**Authors:** Omar Torres-Quesada, Sophie Strich, Eduard Stefan

**Affiliations:** 1 Tyrolean Cancer Research Institute, Innrain 66, 6020 Innsbruck, Austria; 2 Institute of Biochemistry and Center for Molecular Biosciences, University of Innsbruck, Innrain 80/82, 6020 Innsbruck, Austria

**Keywords:** kinases, signaling, mitochondria, kinase inhibitors, cancer, drug off-target effects

## Abstract

Protein kinases take the center stage in numerous signaling pathways by phosphorylating compartmentalized protein substrates for controlling cell proliferation, cell cycle and metabolism. Kinase dysfunctions have been linked to numerous human diseases such as cancer. This has led to the development of kinase inhibitors which aim to target oncogenic kinase activities. The specificity of the cancer blockers depends on the range of targeted kinases. Therefore, the question arises of how cell-type-specific off-target effects impair the specificities of cancer drugs. Blockade of kinase activities has been shown to converge on the energetic organelle, the mitochondria. In this review, we highlight examples of selected major kinases that impact mitochondrial signaling. Further, we discuss pharmacological strategies to target kinase activities linked to cancer progression and redirecting mitochondrial function. Finally, we propose that cell-based recordings of mitochondrial bioenergetic states might predict off-target or identify specific on-target effects of kinase inhibitors.

**Figure:**
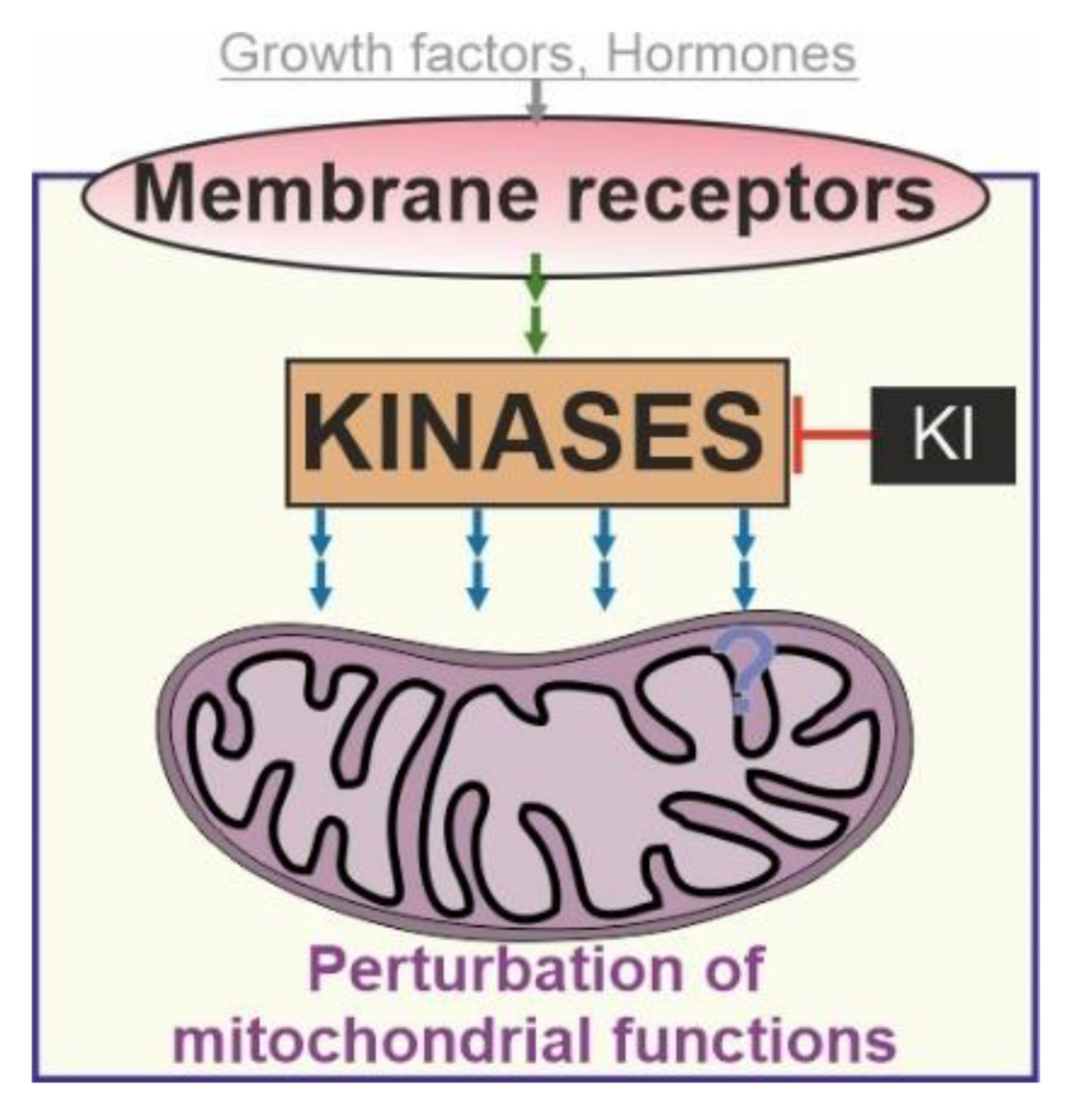

## 1 Introduction

Cells transmit environmental cues through intracellular signaling networks to translate the spatiotemporal input signal into the appropriate cellular response. In numerous signaling pathways post-translational modifications (PTMs) and the formation of diverse molecular interactions (i.e. protein:protein, RNA/DNA:protein and small molecule:protein interactions) take the center stage for adapting cellular functions through alterations of gene expression, cell proliferation and cell energy metabolism (Langeberg, Scott 2015; Nooren, Thornton 2003). Protein kinases are at the heart of numerous signaling pathways. Conventionally, kinases catalyze the transfer of the gamma phosphate of ATP to defined amino acids of their target proteins resulting in a change in enzymatic activity, cellular localization, stability and/or physical interactions. Most human phosphotransferases are serine/threonine kinases through phosphorylating hydroxyl groups of serines and threonines. The others are classified as tyrosine phosphorylating kinases (Ramms et al 2021; Taylor et al 2012b; Ubersax, Ferrell 2007).

Kinase deregulation has been linked to several human diseases, primarily carcinogenesis (Cohen 2002; Ramms et al 2021). Thus, kinases have become the subject of intensive academic and pharmaceutical research. Small molecules targeting decontrolled kinase activities currently are among the most effective drugs in target-oriented cancer therapy (Bhullar et al 2018; Karaman et al 2008; Zhang et al 2009). Kinase inhibitors (KIs) have received FDA approval for the treatment of a collection of malignancies (Roskoski 2019). Much more than 150 kinase-targeted drugs are in late clinical-phase trials and many are in the preclinical stage of drug development. Many of those molecules represent ATP-competitive inhibitors, especially for their use in cancer treatments (Bhullar et al 2018; Hartmann et al 2009).

**Figure 1:**
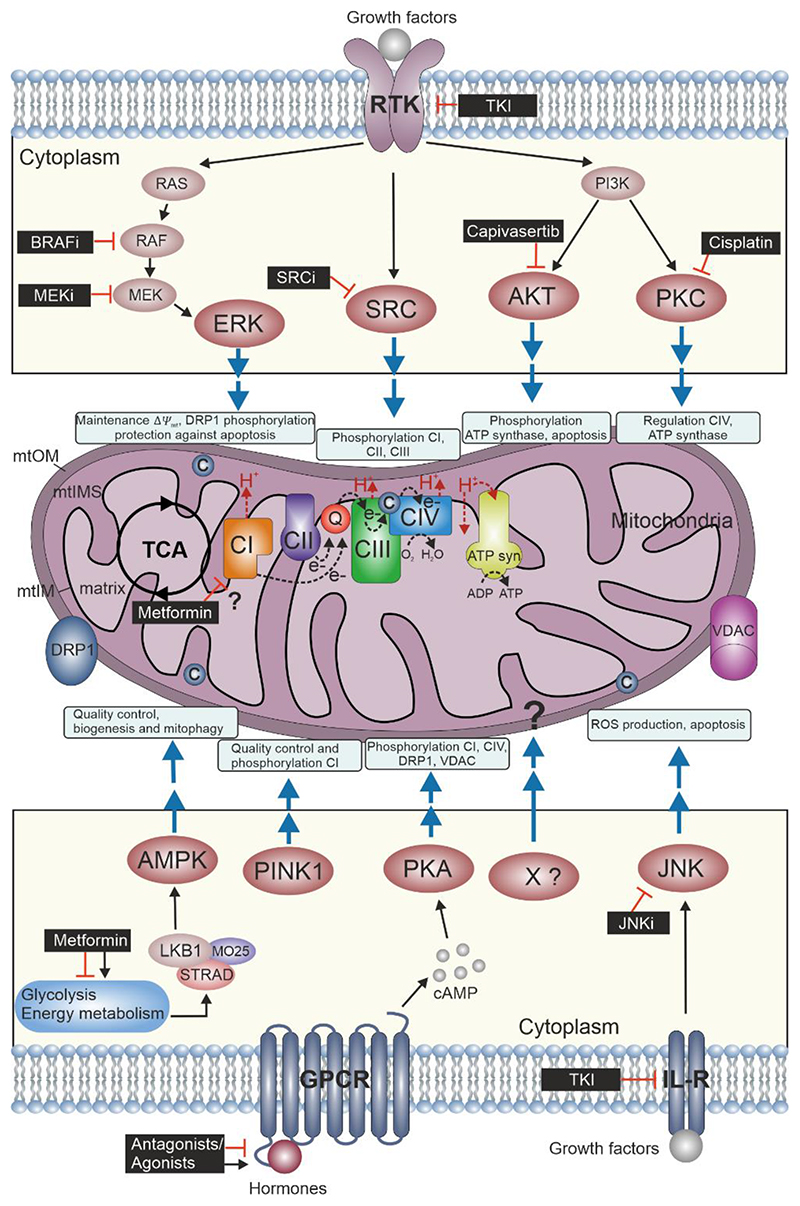
Perturbing the Mitochondrial Kinase Network. Mitochondria consist of four compartments: outer membrane (mtOM), intermembrane space (mtIMS), inner membrane (mtIM) and matrix. The oxidative phosphorylation (OXPHOS) system comprises the ETS and the phosphorylation system (including ATP synthase [ATP syn]) where the reduced fuel substrates coming from the tricarboxylic acid cycle (TCA) and other metabolic pathways are oxidized by electron transfer, chemiosmotic coupling to the phosphorylation of ADP to ATP and intrinsically uncoupling by proton leak and slip, cation cycling, and electron leak. Electrons fuel from NADH-linked substrates to Complex I (CI) and from succinate to CII. These and other electron transfer pathways converge at the Q-junction with further transfer to CIII, cytochrome *c* (c) and CIV, where electrons reduce O_2_ producing H_2_O. The protonmotive force generated is utilized at the ATP synthase to phosphorylate ADP to ATP (Gnaiger et al 2020). Growth factors bind to RTKs and may activate cell type specifically ERK, SRC, AKT and PKA kinases. These might be involved in directly/indirectly influencing activities of the respiratory complexes or mtOM proteins by phosphorylation and control mitochondrial dynamics and bioenergetics. AMPK, PKA and JNK are activated by diverse types of membrane receptors (i.e. GPCRs and interleukin receptors, IL-R, respectively) and control mitochondrial fission, ETS and apoptosis. AMPK and PINK1 are the mitochondrial rheostat for energy state and quality control in response to cellular stress. Kinase inhibitors (black boxes) modulate mitochondrial function by inhibiting kinase activities. The specificity and efficacy depend on cell type, timing and doses of the applied inhibitors which determine the portfolio of kinases (on-target and off-target) that are affected.

Spatial regulation of protein phosphorylation acts as a molecular switch for many regulatory events in signaling pathways that drive cell fate (e.g. cell division, proliferation, differentiation, and apoptosis). Mitochondria have a central role in programmed cell death. For example, phosphorylation of apoptotic and anti-apoptotic proteins influenced by mitochondrial permeability triggers shuttling or release of proteins which directly control cell apoptosis (i.e. phosphorylation of the BCL-2 protein family members, opening of the mitochondrial permeability transition pore and release of cytochrome *c*) (Bononi et al 2011; Kotrasova et al 2021; Niemi, MacKeigan 2013). In this review, we highlight a selection of major kinases which regulate mitochondrial function. Further, we discuss current pharmacological strategies which are based on small molecules/kinase inhibitors to target kinase activities linked to cancer progression showing diverse effects on mitochondria (Figure 1). First, we list a selection of major kinases that participate in modulating mitochondrial dynamics, bioenergetic states and cell fate. Second, we present KI strategies that somehow redirect mitochondria signaling. Third, we emphasize off-target effects of anti-cancer small molecules and how drug-induced effects on mitochondria function may contribute to unintended pharmaceutical responses.

## 2 Protein kinases modulate mitochondria

Mitochondria are the powerhouses of the cell. Besides their central bioenergetic function, mitochondria adopt important roles in cellular signaling and they are central contributors to cell apoptosis, autophagy and cell differentiation (Gnaiger 2020; Rinaldi et al 2018). Deregulated phosphorylation of mitochondrial proteins has been connected with mitochondrial dysfunction. Besides aging diseases such as neurodegeneration, diabetes and cancer have been linked to deregulation of the central ATP producing organelle (Burté et al 2014; Wallace 2012). The current literature has identified at least 30 kinases which have been reported to be functionally linked and/or to phosphorylate mitochondrial proteins (Kotrasova et al 2021; Lucero et al 2019). Thus, we have decided to present a short selection of protein kinases linked to mitochondria which are targets of kinase blockers. We divided our selection based on the mitochondrial functions they modulate.

### 2.1 Kinases modulating mitochondrial biogenesis and dynamics

Mitochondrial dynamics (i.e. fusion, fission and biogenesis) are controlled by a plethora of signaling events. Many extracellular stimuli are sensed by G-protein-coupled receptors (GPCRs) to mobilize second messenger oscillations (Dorsam, Gutkind 2007). The cAMP-dependent Protein Kinase A (PKA) is one of the best-studied examples of a second messenger sensor (cAMP) and allosteric protein complex (Ramms et al 2021; Taylor et al 2012a). Besides its multifaceted functions, macromolecular PKA complexes have been linked to mitochondrial physiology (Rinaldi et al 2018; Torres-Quesada et al 2017). Distinct PKA regulatory subunits are anchored to the outer mitochondrial membrane (mtOM) through the A kinase anchoring protein 1 (AKAP1) (Affaitati et al 2003). PKA substrates compartmentalized at the mtOM are the dynamin-related protein 1 (DRP1) and the voltage-dependent anion channel (VDAC). PKA phosphorylates DRP1 and thus blocks its translocation to the mitochondrial surface to influence mitochondrial fission (Chang, Blackstone 2007). PKA phosphorylates VDAC to modulate the Ca^+2^ uptake and control mitochondrial function (Bera, Ghosh 2001).

Homeostasis control and energy metabolism govern mitochondrial fragmentation, biogenesis, and mitophagy. One of the key sensors of energy homeostasis is the AMP-activated protein kinase (AMPK). AMPK activity increases in dependence of AMP and ADP levels. These are central and direct metabolic cues for adjusting the states of mitochondrial bioenergetics (Herzig, Shaw 2018). In the cytoplasm, AMPK is regulated by an activated trimeric LKB1/STRAD/MO25 complex. As a consequence of high-energy demand AMPK is found at the mtOM for phosphorylating several factors related to mitochondrial fission (i.e. DRP1, MFF), mitophagy and mitochondrial biogenesis (Zhang, Lin 2016). AMPK is a key player in maintaining mitochondrial quality control. In cellular energy stress conditions, mitochondrial populations present different morphologies and dynamics (i.e. changes in mitochondrial membrane potential Δ*Ψ*_mt_, fragmentation or inhibitors of the electron transfer system [ETS]) which activate AMPK feedback loops to further promote mitochondrial fission and autophagy, thus serving as a molecular rheostat of mitochondrial quality (Drake et al 2021; Zhang et al 2018). Another key kinase controlling mitochondrial quality is the PTEN-induced kinase 1 (PINK1). PINK1 mutations are linked to autosomal recessive familial Parkinson’s Disease (PD) (Jones 2010). PINK1 is expressed in mammalian cells and accumulates in specific mitochondria compartments, being found on the inner and outer mitochondrial membrane. PINK1 regulates Parkin, which acts as an E3 ubiquitin ligase. PINK1 recruits Parkin to depolarize mitochondria. While PINK1 is imported and rapidly degraded by mitochondrial proteases with intact membrane potential, PINK1 breakdown is impaired in mitochondria with reduced membrane potential thus serving as a quality control unit for ensuring mitochondrial quality (Wang et al 2020). In addition, PINK1 phosphorylates DRP1. Low levels of PINK1-phosphorylated DRP1 have been found in cells originating from PD patients. This may represent a novel mechanism of regulation of mitochondrial dynamics independently of Parkin (Han et al 2020). Moreover, loss of PINK1 has been linked with Complex I (CI) damage and oxidative stress (Morais et al 2014).

### 2.2 Kinases modulating respiratory functions and ATP production

Components of the mitochondrial ETS and enzymatic systems involved in energy production can be substrates for protein kinases. For example, PKA, when it is localized in the mitochondrial matrix, phosphorylates the NADH:ubiquinone oxidoreductase (Complex I, CI) and the cytochrome *c* oxidase (Complex IV, CIV) (Amer, Hebert-Chatelain 2018). Phosphorylation of the CI (NDUFS4 subunit) has been demonstrated to increase the enzymatic activity and is relevant for its translocation from the cytosol into the mitochondria (De Rasmo et al 2008). In contrast, elevated levels of PKA cause the hyperphosphorylation of several CIV subunits (i.e. COX1, COX4-1 and COX5b) which are associated with decreased Complex IV activity (Prabu et al 2006).

Another relevant kinase circuit which exerts mitochondrial bioenergetic control is the highly conserved phosphoinositide 3-kinase (PI3K)/AKT transduction pathway, which is activated by growth factors. AKT has been shown to be associated with mitochondria and may phosphorylate elements of the ATP-synthase. This molecular event enhances the ATP production in cardiac cells (Yang et al 2013) and plays a role in the inactivation of several pro-apoptotic proteins at the mitochondria for inducing cell survival (Datta et al 1997). Activation of the PI3K pathway is linked to mitochondrial function via Protein Kinase C (PKC). Activated PKC shows colocalization with mitochondria and translocation into the mitochondrial matrix to phosphorylate target proteins. PKC activities are involved in preventing mitochondrial injury upon cellular stress (Lim et al 2016). SRC kinases are key regulators of cell proliferation, differentiation, survival, cell morphology and motility (Parsons, Parsons 2004). SRC kinases are usually cytoplasmic proteins but there is evidence of SRC localization to the mitochondria to act as molecular switch for controlling mitochondrial function (Djeungoue-Petga et al 2019). SRC kinases phosphorylate components of the ETS such as CI subunits (i.e. NDUFV2 and NDUFB10) which increases the levels of respiration. SRC activates succinate dehydrogenase (Complex II, CII) in response to cellular adaptation to nutrient availability (Acin-Perez et al 2014).

### 2.3 Kinases modulating cell fate

Protein phosphorylation controls many aspects of cell fate (i.e. proliferation, differentiation and cell death) and it is often deregulated in pathological conditions. Mitochondria play a central role in cellular outcome and many signaling pathways related to these processes converge in the metabolic organelle (Bononi et al 2011; Niemi,MacKeigan 2013). Receptor-tyrosine kinases (RTKs) represent the second major receptor pathway relevant in cancer signaling, thus we would like to briefly review this cascade in the context of mitochondrial signaling. Growth factors fine-tune mitochondrial activity by redirecting intracellular kinase signaling. RTKs represent a family of precisely controlled receptors that control cell proliferation, cell differentiation, and cell death (Casaletto, McClatchey 2012; Lemmon, Schlessinger 2010). It has been shown that the mitogen-activated protein kinase pathway (MAPK) targets mitochondria displaying relevance for energy metabolism and programmed cell death (Javadov et al 2014).

The gatekeeper kinase MEK activates ERK1/2 by phosphorylation. In this context ERK1/2 activities have been linked to redirect mitochondrial (dys)function, mitophagy, and apoptosis. This may involve the phosphorylation of mitochondrial proteins such as DRP1 and TRAP1, thereby regulating Δ*Ψ*_mt_ and protecting cancer cells from apoptosis (Kotrasova et al 2021).

Another kinase that contributes to adaptions of mitochondrial function and is activated by growth factors is the c-Jun N-terminal kinases (JNK). JNK may have pro-apoptotic but also anti-apoptotic functions at the mitochondria, depending on the cell type and stimulus. For example, JNK is indirectly activated by cytochrome *c* release from mitochondria and it was shown to amplify the ROS generation at CI, contributing to cell death (Shen, Liu 2006; Zheng et al 2017). However, it has been shown that JNK is necessary for IL-3-mediated cell survival via phosphorylation and inactivation of BCL2-associated agonist of cell death (BAD) (Yu et al 2004).

Here, we have listed a selection of major kinases linked to mitochondrial function. It pinpoints the high complexity of mitochondrial-centered kinase signaling pathways which converge at the energetic organelle at different levels. Many aspects of kinase:mitochodria interlinkage are not yet unveiled due to enigmatic effects of non-specific kinase inhibitors on mitochondria.

## 3 Kinase inhibitors affect mitochondrial function

Many anti-cancer therapies are designed to directly target specific molecules to antagonize cancer cell proliferation. Especially KIs targeting mutated kinases have proven the concept of personalized medicine with better outcomes for cancer patients. The specificities of these KIs have been extensively studied as a strategy for drug repurposing and understanding drug resistance mechanisms. Interestingly, it has been revealed that many KIs have unexpected activities on other cellular pathways thus explaining off-target effects (Klaeger et al 2017; Lin et al 2019; Zhang, Loughran 2011).

Specific-spectrum KIs represent one strategy to tackle aberrant cell functions by targeting a single kinase. However, off-target effects of KI affect other cellular functions amongst others e.g. the mitochondrial bioenergetic states (Wynn et al 2011). For example, BRAF inhibitors (BRAFi) (i.e. Vemurafenib, Dabrafenib) have been designed to block the BRAF kinase mutation V600E in melanoma (Karoulia et al 2017; Mayrhofer et al 2020; Röck et al 2019). In contrast, MEK inhibitors target wild-type proteins (Caunt et al 2015). They are combined with BRAFi to reduce emerging resistance mechanisms in the melanoma patients (Dossett et al 2015; Fleischmann et al 2021). Despite the high specificity of mutated BRAF, BRAFi influence mitochondrial function. In melanoma cells, BRAFi treatment drives the intrinsic aerobic glycolytic phenotype inherent in cancer cells to a more oxidative metabolism by increasing mitochondrial OXPHOS and elevating ROS production (Avagliano et al 2020). Other specific KIs targeting the PI3K pathway have been identified to redirect mitochondrial function. For example, AKT inhibitors are proposed to be a new generation of more efficient blockers of cancer (i.e. Capivasertib and Ipatasertib, Phase III clinical trials). They are supposed to target the protective effect of AKT on mitochondria (Martorana et al 2021). However, it is still elusive how the mitochondrial bioenergetic states are altered. In the case of the PKC inhibitor cisplatin, it has been shown that cancer cells treated with this KI undergo apoptotic cell death which is related to enhancing of ROS levels, mtDNA damage and reduced energy production (Choi et al 2015). Kinase blockade of other mitochondria-associated kinases such as JNK and SRC could be beneficial for cancer therapy but the exact impact on mitochondrial respiration has not been evaluated yet.

Besides the discussed KIs, the largest group of kinase blockers used for cancer therapy are the tyrosine-kinase inhibitors (TKIs). They have been established in recent years to become the preferential first- and second-line therapy for many cancer types. TKIs inhibit the binding of ATP to the catalytic binding site of tyrosine kinases. It is of note, that they differ in the spectrum of targeted kinases, the pharmacokinetics and side effects (Hartmann et al 2009; Rodriguez-Hernandez et al 2020). For example, sorafenib and sunitinib target growth factor receptors (i.e. VEGFR, PDGFR). Sorafenib targets around 70 kinases and sunitinib blocks 170 (Karaman et al 2008). TKIs have been reported to cause mitochondrial damage. Effects on the ETS (i.e. CI damage), apoptosis, mitophagy, ROS production, and altered mitochondrial dynamics (mitochondrial fission and fragmentation) are well-described effects related with the mito-toxicity of TKIs (i.e. cardiotoxicity or hepatoxicity) (Rodriguez-Hernandez et al 2020; Vuda, Kamath 2016). These effects are related to off-target drug effects, therefore, the knowledge of kinase inhibition related to physiological and pathological functions which are connected to mitochondria perturbations is necessary to anticipate specificities and drug efficacies.

Other small molecule-related intervention strategies have been reported to produce side effects and to affect mitochondrial function. For example, Metformin is extensively used as therapy for type-2 diabetes patients. Metformin in a pharmacological dose improves mitochondrial respiration by promoting AMPK-dependent mitochondrial fission. In lower doses, metformin has been identified to target CI and, inhibit PKA-dependent CIV activation and the mitochondrial glycerophosphate dehydrogenase (LaMoia et al 2022; Wang et al 2019). Moreover, metformin also indirectly activates AMPK by activation of LKB1 (Foretz et al 2010) thus influencing cellular energy metabolism. This may involve mitochondria but the precise mechanistic mode of regulating mitochondrial bioenergetics remains poorly understood. Other examples of small molecules modulating mitochondrial dynamics (i.e. biogenesis) have been described. This involves, for example the GPCR-agonist formoterol which activates the beta-2 adrenergic receptor pathway, which somehow redirects mitochondrial bioenergetics (Cameron et al 2017).

In conclusion, kinase inhibitors are the drug of choice for treating different types of cancers. Their varying target specificities contribute to a more or less effective blockade of oncogenic signaling events. Off-target effects of small molecules are the major drawbacks of effective therapies. Amongst others, these are related to unwanted effects on mitochondria function which are interlinked with kinase activities and thus are hampered upon small molecule exposure. Therefore, we would like to propose that more systematic profiling of mitochondrial bioenergetic states in the presence and absence of lead molecules or kinase drugs could become an asset for anticipating non-desired off-target drug features.
